# Perspectives for the future in Italy: animal science higher education, employment, and research

**DOI:** 10.1093/af/vfaa022

**Published:** 2020-07-23

**Authors:** Antonella Baldi, Nicolò Pietro Paolo Macciotta, Giuseppe Pulina, Bruno Ronchi

**Affiliations:** 1 Department of Veterinary Science for Health, Animal Production and Food Safety, University of Milan, Milan, Italy; 2 Department of Agriculture, University of Sassari, Sassari, Italy; 3 Department of Sciences and Technology for Agriculture, Forest, Environment and Energy, University of Tuscia, Viterbo, Italy

**Keywords:** animal science, employment, higher education, Italy, research

**Implications**

- In Italy, the courses in animal science include large educational spaces dedicated to practical aspects and internships—both in university experimental farms and in private companies.
- The investment in research and innovation in Italy is lower than in Europe, with an increase driven by a larger number of progressive private/business investments.
- The Italian job market for animal science graduates has excellent opportunities for rewarding careers.
- Italian animal scientists are active in the area of agricultural and veterinary science; however, in the near future, the potential for jobs and research in animal science must consider the outcomes of the SARS-CoV-2 pandemic crisis.

## Introduction

In 2018, the Italian agrifood system reached 140 billion of euros in gross sales (9% of national gross sales) and represents 43% and 3.2 million employed people (13% of overall employment). Animal products represent 27% of gross sales and, in 2018, lost 2.8% in value compared to a 0.9% increase of the entire sector (Istat, 2019). In 2019, Italian agrifood exports increased 7% over 2018, reaching a value of almost 50 billion euros, with excellent performance in the U.S., UK, and Russian markets (Scordamaglia, 2019). Among animal products, milk and dairy sales increased more than 5%, whereas meat and derivatives lost more than 2% (Antonioni, 2019).

**Figure 1.**
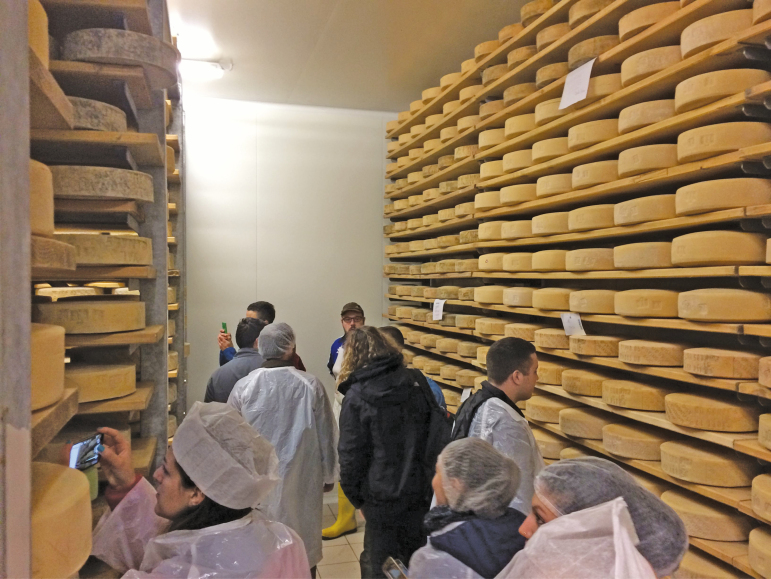
Students observing the production of Parmigiano Reggiano, an example of a Protected Designation of Origin product produced in Italy. This type of specialized product is critical to the sustainability of Italian agrifood production.

Despite this favorable framework, including the global positive trend in Italian food exports, the Italian primary sector remains fragile: the 2010–2013 standard output dropped by 11.5%, while the European Union (EU) gained 7.5%. The ratio of young (<35 yr of age) to old (>65 yr of age) farmers is one of the lowest in the EU (0.11–0.18). The average age of Italian farmers is 57 yr, placing them at the second oldest among the EU countries in rural areas (European Commission, 2017). The information and communication technology sector revolution enacted in Italian agrifood chains will require more and more skilled people. Given all other drivers mentioned above, the employment scenario for highly educated people in the feed, farm, food, and fish Italian animal production systems is rosy! We forecast, even with decreasing turnover, that all current students in animal science and veterinary science (the latter with particular interest to the feed-food chains), will have an excellent chance to find a good job and have a rewarding career considering the increasing production and availability of products bearing the Protected Designation of Origin/Protected Geographical Indication and Indicazione Geografica Tipica designation of animal origin from Italian agricultural production (Figure 1).

### Employment perspectives

Among EU countries, Italy is known for high and persistent youth unemployment. In the age group of 15–29 yr of age, Italians not in education, employment, or training account for 23.4% compared to the EU figure of 12.9%. In addition, the Italian unemployment index in the age group of 25–29 yr of age is 32% compared to 17.2% of the EU28 (Eurostat, 2019). Ten years after graduation, Italian graduates, defined as people who have been in the educational system for ≥16 yr, have lower unemployment rates (12.3%) compared to those people with 13 yr of education (23.2% unemployment rate). Primary school graduates or those people with 8 yr of education have an unemployment rate of 41.6% (Istat, 2019). Five years after graduation, first-level university graduates in agriculture and veterinary sciences (bachelor’s level) have an employment index of 84%, which is lower than the average of their contemporary graduates 88.6% (Figure 1). About 15% of first-level graduates are engaged in independent business activities, such as consulting, entrepreneurial activities or farming, and nearly 50% are employed in a public or private company. They earn less than the average of all Italian first-level graduates (1,297 vs. 1,418 euros/mo of net salary). Table 1 describes the employment, career, and salaries of Italian agricultural graduates and all Italian graduates at both levels. Wage disparity still exists among the sexes with males, first-level or second-level graduates, earning 100 euros/mo more than their female counterparts in agriculture and veterinary sciences (Almalaurea, 2019).

**Table 1.** Comparison between graduates (first and second level) in agriculture and veterinary sciences (SLG/AVS) and all Italian graduates 5 yr after graduation

	Employment (%)		Activity (%)		Salary (€/mo)	
Education level	SLG/AVS	All	Independent	Employed	SLG/AVS	All
First^*a*^	84.0	88.6	15.0	50.0	1,297	1,418
Second^*b*^	83.4	85.6	25.3	43.0	1,382	1,468

^*a*^Equivalent to bachelor’s level.

^*b*^Master’s level.

SLG, starting level graduate; ALG, advanced level graduate.

Regarding the job market, the Italian perspective is not affected by the other EU countries as a result of the free movement of professions guaranteed by the community agreements. Internally, job perspectives for the next 10 yr are predicted to be impacted by turnover in public administration, which provides opportunities for 20% of graduates, private employment for 40%, independent business and entrepreneurial opportunities 30%, and research and development in public or private sector for 10%.

### Italian higher education system in animal science

**Figure 2.**
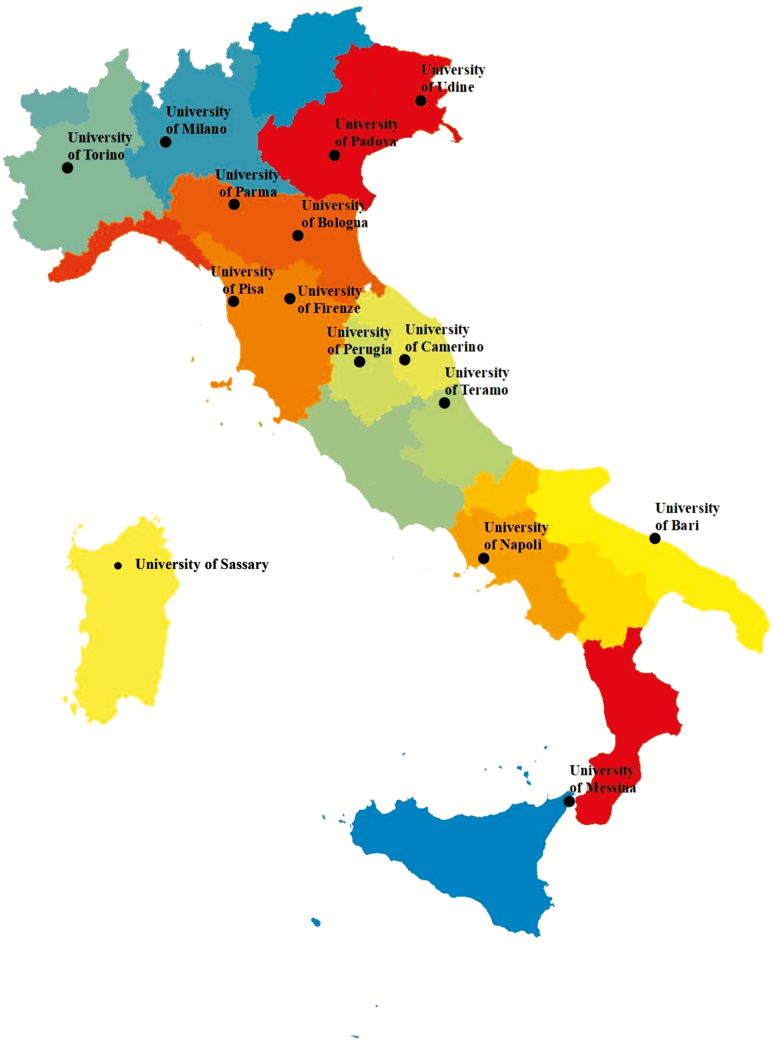
Location of Italian universities offering higher education courses in animal production.

**Figure 3.**
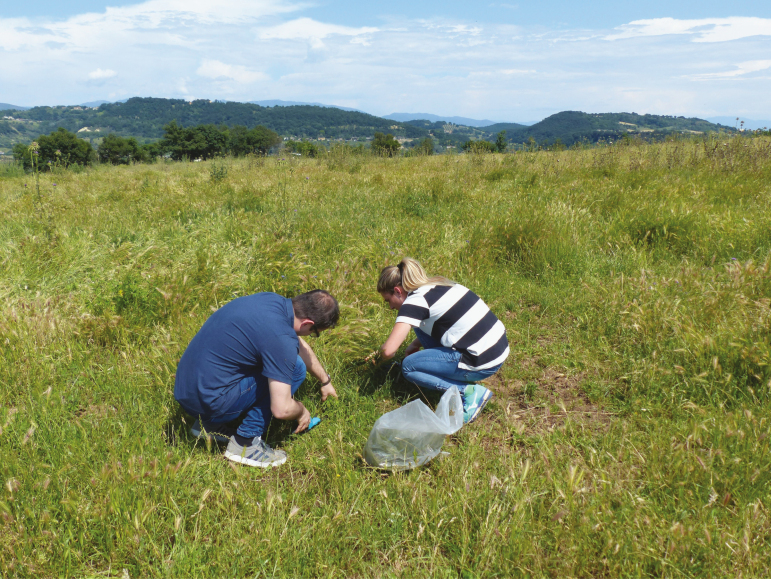
Italian animal science students learning about soil, plant, and pasture health. These skills are critical to providing an effective education in animal production.

Animal Science degree programs in the Italian universities are organized into two cycles, the first cycle consists of 3 yr and the second cycle consists of 2 yr. The 3-yr, first cycle degree course in animal production is currently offered at 14 universities, which are distributed in most regions of Italy (Figure 2). The degree programs are managed either by agricultural sciences departments, by veterinary medicine departments, or jointly by both departments. At a large university campus, a programmed number of students is expected. This means that each state university decides on the number of open seats for students. These seats are determined by the available resources, such as laboratory space, numbers of faculty, and the job market for a degree. In open-access smaller academies, an initial verification is conducted to ascertain the student’s level of preparation for university classes and any formative deficiencies that will impact their performance. This course of study is usually named “sciences and technologies of animal production” and offers a very broad introduction to agro-technical systems to create the knowledge base for the management of livestock farms. However, other courses are also available, such as: “management of sports and companion animals”; “protection of animal welfare”; “wildlife sciences”; “quality of animal production”; “sustainability of animal production systems”; “management of extensive farms” and “hygiene and health in animal farms.” In some universities, there is an international program with the ability to obtain a double degree. The courses in animal production include large educational spaces dedicated to practical aspects and internships—both in university experimental farms and in private agricultural, livestock, processing, marketing, and consulting companies (Figure 3).

The degree thesis usually includes discussion of a project combined with a training internship. A research or experimental thesis is also encouraged. Student experiences at foreign universities through the Erasmus programs (EU student exchange program) are very limited. Animal production students come, in most cases, from scientific or technical-professional secondary education institutions (Almalaurea, 2019). The average time required for a student to obtain the first cycle degree is 4.6 yr. After obtaining the 3-yr qualification, a percentage of students (almost 50%) abandon their studies and look for a job. About 12 mo after graduation, 60% of graduates usually find employment that relates to their educational qualifications. The natural and prevalent continuation of studies is the master’s degree in animal and production sciences.

The second cycle of animal science studies is represented by 16, 2-yr master programs that are currently offered by 15 universities. Courses are managed by departments of agricultural sciences or veterinary medicine or jointly by both departments. There is a wide range of programs at each university. Seven out of sixteen master’s programs have a title of “Animal Science” (or similar) and they are aimed at producing students with a broad range of training that covers several fields of the animal sciences. Other master’s programs are focused on specific topics, such as “food quality and safety” (*n* = 3), “wildlife management” (*n* = 2), “sustainable production” (*n* = 2), “animal welfare” (*n* = 4), and “precision livestock farming” (*n* = 1). Some courses offer a double-degree program. During the master’s program, the student must develop an experimental project that is usually carried out in an internal traineeship at the same university or in a foreign country with the support of the Erasmus program or other international grants. On average, each year, 24 (±13) new students start a Master’s program. About 76% of students earn their master’s title within the 2-yr duration of the program. Five years after graduation, about 75% of the students are employed in a field connected to their qualifications.

All departments are composed of different research units, covering most disciplinary competencies. In animal production, most departments have four disciplines and provide laboratories both for research and teaching. These disciplines are animal genetics, animal nutrition, applied animal science, aquaculture, poultry, and zooculture.

The highest degree of education is the PhD. In 2016, in Italy, there were 12 PhD programs that can be clearly classified as animal science. In the same year, a total of 45 positions in animal science were available to graduates with a PhD. On average, every year, a total of 35 students finish their PhD. In recent years, however, there has been a tendency for a reduced number of positions in animal science that require a PhD. The reduced number of available positions that require a PhD is due mainly to the high costs (about 60,000 euros) for opening a PhD position and the decrease of scholarships available from public funding.

### Research and innovation investment

In Italy, the investment in research and innovation is 1.35% of the GDP (1.97% in Europe) with a slow increase that is essentially driven by a progressive increase of private/business investments and a decrease in public investments. The financial resources allocated by the Italian government for research and development, rather than actual expenditures reported by research and development indicators (OECD, 2019), has decreased by 20% over the past 10 yr. During the same timeframe, research and development expenditures reported by the private sector increased by more than 25%. Despite limited public engagement in supporting research, Italian scientists are quite active in European scenarios in a variety of different fields, including agriculture and animal science. Italian scientists produce a significant number of high-quality publications (as determined by IF and citation indexes) in the area of agricultural and veterinary science that covers about 7.6% of the total number of Italian researchers.

Research and innovation policy are formed both at the national and regional level through the preparation of planning documents and the management of dedicated funds. At the national level, the Ministry of Agricultural, Food and Forestry Policies, the Ministry of Education, University and Research, and the Ministry of Health are the main institutions that deal with agro-food research and they also interface with the EU. In December 2019, the Italian government included in the 2020 budget funding for a new national agency for research as an independent body for the coordination of research funding.

Strategic plans for innovation and research in the food and forestry agricultural sector (RDP 2014–2020) supported the following topics:

- Sustainable increase in productivity, profitability, and efficiency of resources in agro-ecosystems;
- Climate change, biodiversity, soil functionality, and other ecological and social services of agriculture, quality and typicality of agricultural products, food safety, and healthy lifestyles;
- Sustainable use of biological resources for energy and industrial purposes; and
- Development and reorganization of the knowledge system for the agricultural, food, and forestry sector.

At the regional level, agricultural research is regulated by specific rules, regarding innovation development and transfer services. Regional administrations have full autonomy of action. At the regional level, the investments in zootechnical research is about 25% of the total investment in agricultural research (Macrì, 2017; research financed by the regions 2017). If the main issues supported at a territorial level in recent years are examined, the most frequently pursued objective has been that of developing new products and processes for improving product quality. A further goal has been the achievement of innovations aimed at decreasing production costs, management of natural resources, and enhancing biodiversity and the health and well-being of farm animals (with particular focus on alternatives to antimicrobials).

In order to strengthen the area of research and innovation, the Ministry of Research and University, since 2012, has supported the creation of a national cluster of agrifood, a recognized association of companies, universities, research centers, and territorial representations, for the development and enhancement of national technology in the agrifood area and the promotion of life-long learning initiatives.

**Figure 4.**
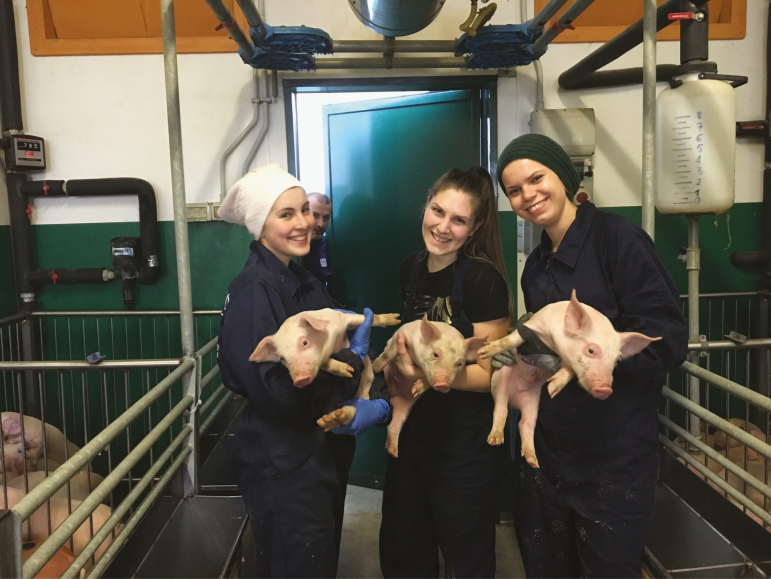
Animal science students working with pigs in a hands-on laboratory. Animal handling skills will always be an essential part of an animal science education.

In a perspective that aligns with the 2030 Agenda on sustainability goals, new challenges in the field of research and innovation in animal science must support sustainable competitiveness and encourage the development of healthy and safe breeding systems to secure food safety (goal 2). Interventions aimed at encouraging the supply of environmental services by livestock production systems, reduction of greenhouse gas emissions, definition of integrated strategies for disease prevention, and control activities (goal 15) are priorities. Animal scientists will be asked to repurpose food waste not intended for human consumption for feed use (goal 13). Innovation must consider not only technical-production elements but must also favor improvement of the organizational and management components of livestock production (Figure 4). Precision farming and feeding technologies, as well as new techniques in animal breeding and reproduction, are increasingly essential to compete effectively in the global market of agrifood products.

## Future Considerations

The direction and types of training needed for graduates in animal science are difficult to forecast. The changing needs associated with the information and communication technology and biotechnology sectors makes forecasting specific careers a difficult task. To adapt to a dynamic environment, the human capital in livestock applied science and basic disciplines, as well as soft skills, will be reinforced in animal science graduate courses. Training in disciplines such as biology/biodiversity, process engineering, economics, computer science, and communication will be required. In a world in which tomorrow’s jobs have not been invented yet, students must be trained as life-long learners who have been trained in competency-based, challenge-oriented international programs. The future animal science educational programs should change not only in terms of innovative content but also in terms of teaching methods. In this direction, the new generation of animal science courses must use new technological innovations and change their approaches in defining the curriculum content. Short continuous massive open online courses will be continuously generated for the maintenance and improvement of the graduates’ skills necessary for continuous transformation of animal production chains. Additional critical skills students will require include soft skills such as communication, networking, leadership, advising, planning, and organizing. Development of these skills must be prioritized in programs to create a well-trained group of professionals. Only this integrated approach will allow students to develop a modern range of knowledge related to the livestock value chain and to build confidence in the subject and in themselves.

This scenario has undergone an incredible acceleration due to the COVID-19 pandemic. This paper was written before the SARS-CoV-2 coronavirus reached Italy and Europe in full force in early 2020. During recent months, universities adapted quickly and developed distance learning solutions for education and training programs to meet social distancing requirements in an attempt to slow the spread of the virus and minimize the impact on the health care system. This experience makes it clear that nothing will return as before once the crisis has been overcome. The impact of the COVID-19 pandemic on the Italian zootechnical system and the food supply chain will be a major topic of debate among scientists, professionals, and stakeholders for many months to come. Analysis of the short-and medium-term effects of the pandemic on the food production system must be given high priority among the many challenges animal scientists must face in the near future. Additionally, the increased demand for sustainable animal products with the increasing availability of nonconventional foods and the need for sustainable feed production systems creates a challenge for the animal scientist. Food accessibility and economical production must be balanced with the emerging interest by consumers of animal products to ensure ethical animal production systems.

## Conclusions

The future of research and training programs in animal science departments in Italy is as much of a challenge as animal scientists have ever experienced. The limitations in free movement of people and products caused by the SARS-CoV-2 pandemic will have a long-lasting impact on animal production and animal science. Given the growing demand for ethical food production systems by the millennial generation, animal science graduates must be nimble and flexible enough to rapidly design and implement new strategies for the production of animal-sourced foods. We must prepare future animal scientists with as much of a broad and diverse background of knowledge and skills as possible that will enable them to establish cross-disciplinary approaches among animal, human, and environmental health in a “one-health” approach. In addition, future animal scientists will need to be equipped with new models of animal production systems, coupled with smart working skills and information and technology tools, efficient and safe use of resources, and robust communication capabilities to meet societal demands for animal-sourced foods.

## About the Authors

Antonella Baldi is professor in animal nutrition at the University of Milan, where she also serves as Vice Rector for international relationships. Her main research interests are the role of animal nutrition and welfare in the production of high quality and healthy food of animal origin, in particular milk. She studied the biological mechanisms underpinning the function and development of the mammary gland in dairy animals. She was involved in the European Cooperation in Science and Technology and was the Italian delegate on the Food and Agriculture Committee. She was Vice President of the Italian Animal Science and Production Association. and was a member of the EAAP Council. She is Editor of the Product Quality, Human Health and Well-Being section of *Animal* since the foundation of the journal in 2007.

Giuseppe Pulina, PhD, is professor of animal science at the Department of AGRARIA, University of Sassari (Italy). He teaches ethics and sustainability of animal production and ethics of the scientific research for graduate students. His research focuses on dairy sheep and goat feeding and nutrition, mathematical modeling, milk quality, environmental impact of livestock, genetic improvement, and animal welfare. He was President of the National Conference of AGRARIA (former conference of the Deans of Agriculture); Vice President of the Association of Scientific Societies of Agriculture, and President of the Association for Science and Animal Production, of which he is currently President Emeritus. He is an ordinary member of the Accademia dei Georgofili of Florence, honorary member of the Pesaro-Urbino Academy of Agriculture, and a corresponding member of the Agricultural Academy of Bologna. He is coordinator of Assalzoo Scientific Committee and President of Carni Sostenbili (Sustainable Meat) Association.

Bruno Ronchi is professor of animal nutrition and feeding at Tuscia University, Viterbo, Italy. His research mainly concerns studying effects of heat stress on domestic ruminants; effects of exposure to chemical environmental contaminants on animal health and on safety of animal products; and evaluation of chemical composition and nutritional value of forages and concentrates for animal feeding. From 2007 to 2009, he was Director of the Tuscia University Experimental Farm; Dean of the Faculty of Agricultural Sciences (2009–2011); member of Administration Council (2009–2011); and Quality Unit Coordinator (2017–present). He was President of the Animal Science and Production Association (2013–2019) and has been a council member of the European Federation for Animal Science since 2018.

Nicolò Macciotta is professor of animal breeding and genetics at the Department of Agricultural Science of the University of Sassari, Italy. He is Chair of the Bachelor and Master programs in animal science at the University of Sassari. He is currently President of the Italian Animal Science and Production Association. His fields of expertise are animal breeding, genomics, statistics and modeling, and livestock biodiversity.
